# Epigenetic Crosstalk between the Tumor Microenvironment and Ovarian Cancer Cells: A Therapeutic Road Less Traveled

**DOI:** 10.3390/cancers10090295

**Published:** 2018-08-30

**Authors:** Yuliya Klymenko, Kenneth P. Nephew

**Affiliations:** 1 Cell, Molecular and Cancer Biology Program, Medical Sciences, Indiana University School of Medicine, Bloomington, IN 47405, USA; 2 Department of Chemistry and Biochemistry, Harper Cancer Research Institute, University of Notre Dame, South Bend, IN 46617, USA; 3 Department of Cellular and Integrative Physiology and Obstetrics and Gynecology, Indiana University School of Medicine, Indianapolis, IN 46202, USA; 4 Indiana University Simon Cancer Center, Indianapolis, IN 46202, USA

**Keywords:** ovarian cancer, epigenetics, tumor microenvironment, DNA methylation, histone modifications, chromatin remodeling, non-coding RNAs

## Abstract

Metastatic dissemination of epithelial ovarian cancer (EOC) predominantly occurs through direct cell shedding from the primary tumor into the intra-abdominal cavity that is filled with malignant ascitic effusions. Facilitated by the fluid flow, cells distribute throughout the cavity, broadly seed and invade through peritoneal lining, and resume secondary tumor growth in abdominal and pelvic organs. At all steps of this unique metastatic process, cancer cells exist within a multidimensional tumor microenvironment consisting of intraperitoneally residing cancer-reprogramed fibroblasts, adipose, immune, mesenchymal stem, mesothelial, and vascular cells that exert miscellaneous bioactive molecules into malignant ascites and contribute to EOC progression and metastasis via distinct molecular mechanisms and epigenetic dysregulation. This review outlines basic epigenetic mechanisms, including DNA methylation, histone modifications, chromatin remodeling, and non-coding RNA regulators, and summarizes current knowledge on reciprocal interactions between each participant of the EOC cellular milieu and tumor cells in the context of aberrant epigenetic crosstalk. Promising research directions and potential therapeutic strategies that may encompass epigenetic tailoring as a component of complex EOC treatment are discussed.

## 1. Introduction

Epithelial ovarian cancer (EOC), a histopathologically, morphologically, and molecularly heterogeneous group of neoplasms [1], is the leading cause of gynecological malignancy-related deaths in women, with >14,000 deaths in the United States (US) and ~152,000 deaths worldwide yearly [2,3,4]. Most women have vastly disseminated intraperitoneal disease at the time of diagnosis contributing to a five-year survival rate of only 30% [5]. Development of multidrug resistant and essentially incurable tumor recurrence in the majority of patients after initial good response to standard platinum/taxane-based chemotherapy are also significant factors contributing to this deadly disease [6,7].

### 1.1. Tumor Microenvironment (TME) Associated with Ovarian Neoplasms

**Figure 1:**
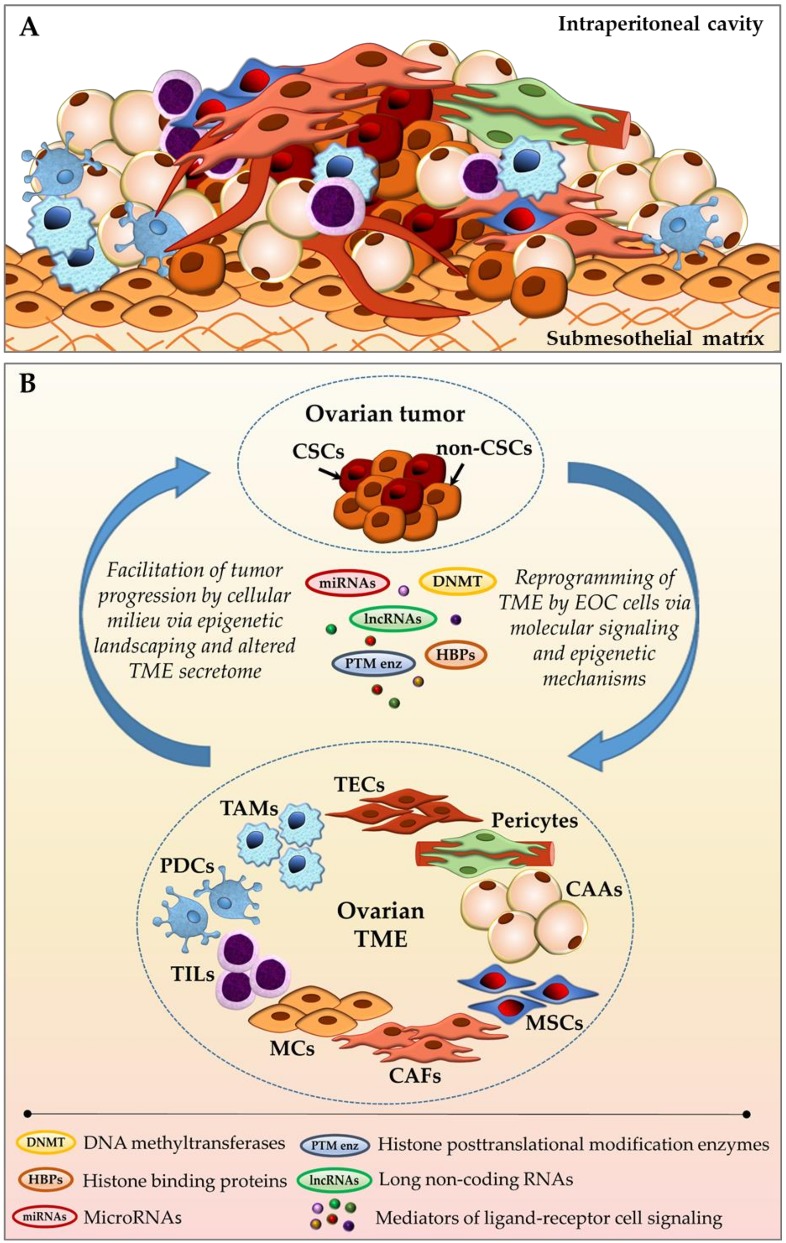
Ovarian tumor-stroma bidirectional crosstalk. (**A**) Schematic representation of cellular diversity within the complex ovarian tumor bulk; and, (**B**) Reciprocal communication between ovarian cancer cells and intraperitoneally residing cancer-associated cellular milieu components via molecular signaling pathways and epigenetic regulation. CAAs—cancer-associated adipocytes; CAFs—cancer-associated fibroblasts; CSCs—cancer stem cells; EOC—epithelial ovarian cancer; MCs—mesothelial cells; MSCs—mesenchymal stem cells; PDCs—plasmacytoid dendritic cells; TAMs—tumor-associated macrophages; TECs—tumor-associated endothelial cells; TILs—tumor-infiltrating lymphocytes; TME—tumor microenvironment (see main text for details).

EOC initiation results from accumulation of genetic mutations and epigenetic changes resulting in malicious transformation of epithelial cells, stem cells, or transient metaplastic regions at the primary site, either ovary or the fallopian tube fimbriae [8,9,10,11,12,13,14,15,16,17,18]. While lymph node and hematogenous metastasis of ovarian cancer have been reported in human EOC cancer and/or model systems [19,20], the current consensus is that expansion of ovarian neoplastic masses occurs primarily via transcoelomic route, including the direct exfoliation of anoikis-resistant cancer cells and multi-cellular clusters from the original tumor, ascitic fluid-facilitated intraperitoneal dissemination, subsequent mesothelial adhesion and retraction, submesothelial extracellular matrix invasion, and ultimate establishment of secondary lesions in peritoneum-sheathed surfaces and organs [18,21,22,23]. During this metastasis process, ovarian cancer cells are confined to and nurtured by the complex host intraperitoneal cellular milieu, encompassing cells co-existing within the tumor bulk, freely available in ascitic effusions, and residing in peritoneal and adipose tissues—fibroblasts, mesothelial cells, adipocytes, infiltrating lymphocytes, macrophages, plasmacytoid dendritic cells, mesenchymal stem cells, and others (Figure 1) [24,25,26,27,28,29]. Both EOC and host non-cancerous cells secrete a plethora of bioactive soluble constituents—proteins, growth factors, phospholipids, hormones, cytokines—into the extracellular space and malignant ascites [23,27,30,31,32,33,34,35,36,37,38,39,40,41,42,43,44], collectively generating a dynamic intraperitoneal TME that mediates ovarian cancer development, metastatic progression, and therapeutic response through receptor-ligand (autocrine, paracrine, endocrine) signaling, contact-dependent (juxtacrine) cell signaling, as well as epigenetic regulation (Figure 1B).

### 1.2. Basic Epigenetic Mechanisms at a Glance

**Figure 2:**
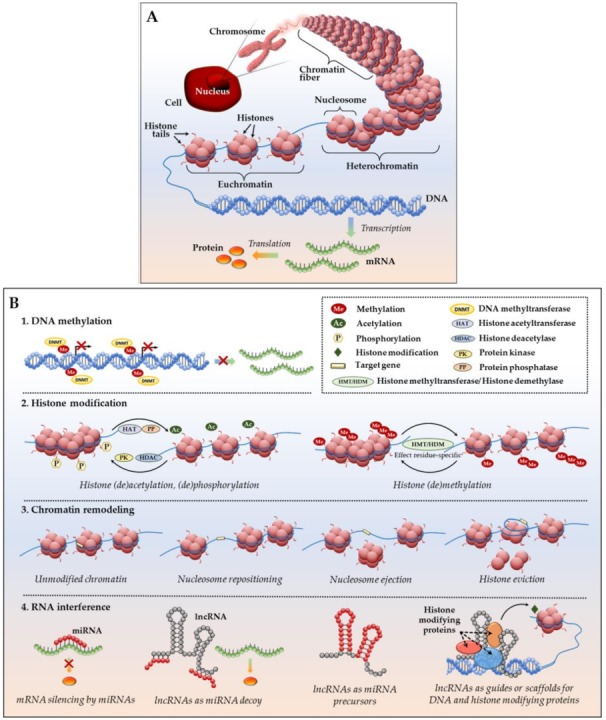
Epigenetic regulation of gene expression. (**A**) DNA packing in a eukaryotic cell: a DNA molecule (chromosome) located inside the cell nucleus is composed of chromatin fibers which are made of nucleosomes—histone octamers wrapped by a DNA helix. Condensed chromatin (heterochromatin) is transcriptionally silent; loosely packed euchromatin allows access to DNA and active transcription into mRNA, followed by translation into protein; (**B**) Major epigenetic mechanisms of gene expression include: (1) DNA methylation; (2) histone modifications, such as histone acetylation, methylation, phosphorylation, etc.; (3) chromatin remodeling, including nucleosome sliding, nucleosome ejection and histone eviction; and, (4) mRNA interference with miRNAs and lncRNAs (see main text for details).

Epigenetic modifications are heritable alterations in gene expression (activation or suppression) that occur as a result of perturbed chromatin organization and altered gene accessibility for transcriptional machinery in the absence of changes to the DNA itself [45]. Additionally, epigenetic mediation encompasses the modulation of gene expression at the posttranscriptional level via altered mRNA translation into protein (Figure 2). Fundamental epigenetic regulatory mechanisms include:

1. DNA methylation—addition of methyl groups to DNA CpG sites without altering DNA nucleotide sequence. Methylation occurs by means of enzymes called DNA methyltransferases (DNMTs), which place methyl groups on symmetric cytosine residues in double-stranded CpG sites [46,47]. Hypermethylation of CpG islands (nucleotide sequences enriched for CpG sites) in the promoter regions of tumor suppressor genes (TSGs) and growth regulatory genes prompts gene silencing [46,47] as attached methyl groups physically block binding of transcription factors to the gene promoters. Alternatively, dense DNA methylation interferes with the proper nucleosome positioning [48]. Within the DNMT family (including three active enzymes, DNMT1, DNMT3a, and DNMT3b), DNMT1 exhibits high preference for hemimethylated DNA (in which one of two complimentary DNA strands already possess attached methyl groups), and is therefore responsible for so called “maintenance methylation” [49,50]. DNMT3a and DNMT3b are primarily responsible for the “de novo” methylation of previously unmethylated CpG regions [51,52], but both of these methyltransferases have been shown to carry out maintenance methylation as well [53]. Importantly, in human neoplastic cells, it has been shown that DNMT1 provides both de novo and maintenance DNA methylation of TSGs [54,55,56]. The demethylating agents (or hypomethylating agents (HMAs) that inhibit these enzymes (azacitidine or AZA; decitabine or DAC; SGI-110 or guadecitabine) are discussed below).
2. Histone modifications—various posttranslational modifications (PTMs) at histone protein N-terminal tails, which impair proper interactions between adjacent nucleosomes to affect the compact packing of the chromatin and impede the binding ability of other factors/enzymes that are involved in gene transcription [57,58]. The most common and well-characterized PTM, histone acetylation, is a dynamic, reversible process in which positively charged histone lysine residues are neutralized via the addition of acetyl groups by histone acetyltransferases (HATs), resulting in the attenuation of bonds between negatively charged DNA string and a histone complex. In the reverse reaction, deacetylation, enzymes histone deacetylases (HDACs) remove acetyl groups, and reinforce positive charge of the lysines, securing compact wrapping of DNA around histones [59,60]. Similarly, histone (de)phosphorylation utilizes protein kinases and phosphatases to attach or remove negatively charged phosphate groups, respectively, influencing chemical attraction between DNA and histone tails (reviewed in [60,61]). Histone (de)methylation—addition/removal of methyl groups by histone-specific methyltransferases and demethylases—can either activate or silence gene transcription. Remarkably, the functional consequences of each histone (de)methylation event depend on the histone, amino acid and residue methylated, degree of modification (mono-, di- or tri-methylation), and attraction of additional function-specific protein cofactors to the site, as well as existence of other methyl or acetyl groups in close proximity (reviewed in [62]). Comprehensive analyses of currently known histone PTMs, including those less common (ubiquitylation, sumoylation, deamination, etc.), their functional outcomes and complex interplay between the DNA methylation and histone modifications have been recently published [63,64].
3. Chromatin remodeling—rearrangement of chromatin organization through complete or partial nucleosome repositioning and altering gene access for transcription. Chromatin remodeling can occur via nucleosome sliding (movement of the core histone octamer nexus across DNA segment with no evident disintegration of the octamer itself), nucleosome ejection (nucleosome segregation from the chromatin chain), or histone eviction (removal of histone H2A/H2B dimers from the DNA-associated nucleosome, sometimes with an alternative histone replacement) [65,66,67]. These processes are mediated by a number of ATP-dependent chromatin remodelers with high binding affinity to modified core histone tails, as well as transcriptional enzymes, which are extensively described in [65,66,67]. In particular, ARID1A and SMARCA4 are prominent chromatin remodeler examples in ovarian cancer. ARID1A is frequently mutated in ovarian clear cell (~50%) and low grade ovarian endometrioid (30%) carcinomas [68,69]. Most interestingly, tumors with ARID1A mutations acquire sensitivity to pan-HDAC inhibitors, thus making ARID1A-bearing cancers attractive for HDAC-based therapy [70]. SMARCA4 is frequently (over 90%) mutated in ovarian small cell carcinomas of the hypercalcemic type [71,72], however, to our knowledge, the first case of a germline SMARCA4 mutation in a patient with HGSOC was recently reported [73]. Further investigation on the role and clinical applicability of SMARCA4 and ARID1A in HGSOC is warranted [74,75].
  Altogether, the three epigenetic mechanisms that are described above work closely to mediate DNA (un)coiling around the core histones and ensure dynamic chromatin reassembly between heterochromatin (condensed or closed, silent) and euchromatin (loose or open, transcription-permissive) states.
4. Non-coding RNA interference—a group of epigenetic regulatory mechanisms that involves microRNAs (miRNAs; miR) and long non-coding RNAs (lncRNAs). MiRNAs are short (~22 nucleotides) non-messenger RNAs that act primarily at a posttranscriptional level by base pairing with their complimentary mRNA targets to alter mRNA translation into protein [76]. Remarkably, one miRNA may complement a variety of mRNAs, whereas the same mRNA transcript might be a target of multiple miRNAs. Additionally, miRNAs may act as mRNA destabilizers causing poly-A-tail shortening [77] or interfere at the gene transcriptional level by means of PTMs (e.g., initiation of histone H3 lysine^9^ methylation with RNA interference machinery, followed by DNA methylation and gene transcription repression) and heterochromatic silencing [78]. LncRNAs are long (>200 for up to a hundred thousand nucleotides) non-messenger RNA that execute epigenetic regulation via several mechanisms: engage in post-translational histone modifications through association with chromatin-modifying proteins as an obligatory active player in the complex or as a scaffold that brings different protein complexes in close vicinity for proper functioning; serve as endogenous competitors to mRNA by base pairing with miRNAs and uncovering mRNAs for effective protein translation; or, serve as precursor RNAs for miRNAs (all mechanisms are detailed in [79,80,81]).

The importance of epigenetic dysregulation for cancer progression cannot be understated, as various aberrant epigenetic modifications trigger activation of oncogenes, repression of tumor suppressor genes, and altered transcription of protein-encoding genes that are collectively responsible for proper expression of signaling proteins that are necessary for physiological cell life span and functions. In the light of significant advances in technical tools and the rapid emergence of large “omics” data, the current knowledge in the field of cancer epigenetics and understanding their translational potential have vastly expanded. Multiple elegant reviews illustrate a wealth of currently available data on cancer epigenomics [46,82,83,84,85,86,87]. In the context of EOC specifically, several recent works highlight major epigenetic players—DNA methylation [88,89,90], histone PTMs [88,89], miRNAs [88,89,91,92], lncRNAs [93,94], and discuss current and potential therapeutic implication strategies and challenges [85,95,96,97,98].

## 2. Epigenetic Crosstalk between EOC Cells and TME Cellular Components

The biological importance of TME as a reactive platform orchestrating diverse aspects of tumor initiation, evolvement, metastatic progression, altered immune response, development of therapeutic resilience, and cancer recurrence is unquestionable and continuously reaffirmed, as highlighted in a multitude of studies [99,100,101,102,103,104]. Given the unique ovarian cancer intraperitoneal TME and the emerging evidence of benefits from epigenetic-targeted therapeutics, we systematize epigenetic dysregulations linked to EOC progression from the perspective of complex reciprocal relationship between ovarian tumor cells and tumor-associated microenvironmental non-malignant cell setting. These interactions will allow for researchers to evaluate, comprehensively, the potential for exploiting specific epigenetic vulnerabilities in both cancerous and TME cells as molecular biomarkers, prognostic indicators, and complementation to conventional chemotherapy and TME-targeted interventions.

### 2.1. Cancer-Associated Fibroblasts (CAFs)

CAFs are traditionally abundantly present within the tumor stroma (Figure 1A). They serve a tumor-supporting role via remodeling of extracellular matrix, a scaffold for the tumor bulk. CAFs perform endocrine/paracrine communication with the surrounding tumor and other stromal components via the excretion of a variety of growth factors and chemokines, hence contributing to tumor growth, immune response, angiogenesis, chemoresistance, and cell stemness; furthermore, CAFs differentiate into other cell types, as comprehensively discussed elsewhere [105,106]. CAFs exhibit substantial heterogeneity, which is attributed to existence of multiple proposed CAF origins, including reprogramming of the normal resident fibroblasts, conversion from adipocytes, endothelial or epithelial cells, or differentiation of the bone marrow derived mesenchymal or hematopoietic stem cells (summarized in [107]). Gene expression analysis of CAFs purified from different types of cancers, including ovarian carcinomas, revealed normal genotype, and acquisition of somatic mutations is considered to be an extremely infrequent event [108,109,110]. Hence, epigenetic changes likely play an essential role in CAF regulation.

Studies have reported altered DNA methylation status of genes (either at the gene promoter regions or global) in stromal fibroblasts dissected from various cancer tissues that corresponded to methylation profiles that are found in adjacent malignant cells [111,112]. In turn, as was shown by Mathot et al. for breast cancer [113], malignant cells maintained in the presence of CAF soluble mediators exhibit a wide gene upregulation pattern (372 genes upregulated total in the study) epigenetically modulated via DNA hypermethylation that could be reversed by the DNMT inhibitor decitabine. Pistone and team [114] demonstrated through global transcriptome and DNA methylation analysis that CAF-secreted factors trigger combinatorial DNA hyper/hypomethylation changes, collectively responsible for epithelial-to-mesenchymal transition (EMT) and stemness phenotype in prostate cancer cells. Albrengues and colleagues [115] reported the transformation of fibroblasts into pro-invasive CAFs in response to a cytokine termed leukemia inhibitory factor (LIF), which triggers the continued activation of JAK1/STAT3 signaling pathway via the histone acetylation of STAT3, followed by the activation of DNMT3b, methylation and abrogation of the Src homology region 2 domain-containing phosphatase-1 (SHP-1), and ultimate sustained phosphorylation of JAK1. The process is chaperoned by DNMT1, whereas the inhibition of DNMTs and JAK signaling abolishes the CAF phenotype [115].

Noteworthy, even brief transitory communication between cancer cells and normal fibroblasts leads to an elevated release of transforming growth factor beta 1 (TGF-β1) by activated fibroblasts in response to cancer cell presence [116]. This may be of particular relevance in the context of ovarian cancer epigenetic regulation, as TGF-β is known to catalyze global DNA hypermethylation alterations in EOC cells, promoting EMT and metastasis [117]. Moreover, Cardenas and co-workers found that TGF-β enhances both expression and enzymatic activity of DNMT-1, -3a, and -3b in EOC cells [117]. Importantly, the hypermethylation effect and EMT may be abrogated by treatment with DNMT inhibitors [117]. On the other hand, TGF-β assists in the reprogramming of CAFs from other cell types [118] and it favors further aggressive invasion of HGSOC through TGF-β-induced secretion of prometastatic mediators by CAFs [118,119]. Collectively, these data suggest formation of a constitutively active positive feedback loop in EOC, where initial fibroblast-malignant cell interaction triggers secretion of TGF-β by stromal cells, followed by EMT-associated global epigenetic alterations in EOC cells, as well as promotion of CAF phenotype and secretory activity, which closes the cycle and further aggravates EOC. In this scenario, the combined usage of demethylating agents and drugs targeting TGF-β signaling requires further investigation.

To our knowledge, posttranscriptional histone modifications in EOC-associated CAFs have not been directly addressed, whereas similar studies in the context of other cancer types are very limited. The histone mark H3K27me3 in breast CAFs was shown to be reduced, along with decreased expression of enhancer of zeste homolog 2 (EZH2), a methyltransferase that is responsible for the H3K27me3 histone mark [120], resulting in promotion of cancer cell invasion by CAFs via upregulated thrombospondin type 1 motif 1 [121]. In EOC, however, EZH2 is commonly upregulated [122], and thereby, such a mechanism is not likely. Undoubtedly, studies are needed in elucidating histone PTMs possibly taking place during malignant cell-CAF interplay.

Malignant TME-driven implication of miRNAs in CAF phenotype and functioning has been dissected for various cancer types (breast [123], cervical [124], gastric [125], prostate [126], colorectal [127,128], and bladder [129]), including EOC. In an elegant gain/loss-of-function study employing the co-transfection of multiple miRNA mimics and inhibitors, Mitra et al. [130] discovered a combination of three dysregulated miRNAs (upregulation of miR-155 plus repression of miR-31 and miR-214) that are capable of converting quiescent fibroblasts into ovarian CAFs with extensive expression of chemokines, in particular, direct target of miR-214 CCL-5 (C-C motif ligand 5), CXCL-10 (C-X-C motif ligand 10), CCL-7 and CCL-8, among others, accompanied by the substantial augmentation of ovarian tumor growth. The respective reverse experiments have shown the restoration of wild-type fibroblast phenotype and alleviation of ovarian tumor growth in co-culture [130], suggesting these miRNAs as novel therapeutic targets for halting EOC progression by means of manipulating stromal signals.

Zhao and coworkers [131] have recently discerned prognostically unfavorable upregulation of lncRNA LINC00092 in EOC cells which is induced by high CXCL-14 (C-X-C motif ligand 14) chemokine expression in ovarian CAFs. Expressed LINC00092 binds to a glycolytic enzyme and it facilitates glycolysis in EOC cells, boosts metastatic activity, and reciprocally supports pro-active CAF phenotype [131]. A differential expression analysis of CAFs purified from 67 high grade serous ovarian carcinomas (HGSOC) and 10 normal ovarian fibroblast samples identified 39 divergently expressed lncRNAs (out of 1970 lncRNAs total analyzed) in HGSOC CAFs in comparison with normal fibroblasts [132]. Subsequent context-specific regulatory network construction and pathway analyses have linked the upregulation of seven lncRNAs (FLJ39739, GAS5, H19, LOC100499466, MALAT1, NEAT1, and TUG1) and downregulation of four lncRNAs (CASC2, DLEU2, HCG18, and LOC100133669) to the furtherance of metastasis-associated pathways in HGSOC [132]. Mechanistic insight on the interaction of these differentially expressed lncRNAs in CAFs with proteins/pathways is essential for the further translation of these findings into diagnostic and/or therapeutic strategies.

### 2.2. Cancer-Associated Adipocytes (CAAs)

One of the distinguishing characteristics of the ovarian cancer TME is tumor cells that are in close vicinity to adipose tissue at all stages of development and metastasis. Adipose tissue exists at the site of the primary tumor (ovarian fat pad, mesosalpingeal/mesoovarian adipose layers) and around the intra-abdominal cavity in the form of substantial omental adipose nodes, large adipose bundles in the mesentery (both serve as prevalent EOC metastasis locations), as well as smaller fat depots in the parietal and diaphragmatic peritoneum (Figure 1A). It is now commonly accepted that adipose tissue is a highly communicative metabolic and secretory organ. Aside from functioning as energy (lipid) storage, it produces adipokines, metabolic substrates, growth factors, hormones, and immune mediators, and it contains other stromal components, such as fibroblasts, stromal vascular fraction, macrophages and other immune cells, nerve tissue, and extracellular matrix [133]. In presence of malignant setting, normal adipocytes rapidly acquire a highly active phenotype (CAAs) and respond by metabolic and secretory profile changes, causing pro-inflammatory, pro-invasive, proliferative, and radio- and chemoresistance effect on cancer cells [28,134,135,136,137,138,139].

There is accumulating evidence for tumor-adipocyte epigenetic interactions. During maturation, murine preadipocytes demonstrate substantial upregulation of miR-17-92 cluster, which further stimulates adipocyte differentiation via negative targeting of tumor-suppressor Rb2/p130 and is known to boost cell proliferation in various cancers [140]. In breast cancer (another type of adipose tissue-rich neoplasm), the transition of adipocytes into inflammatory CAAs in the vicinity of malignant milieu is dependent on miRNA regulatory mechanism. In particular, in the presence of breast cancer cells, reprogramming CAA increase expression of miR-5112, which suppresses the translation of Cpeb1, a negative regulator of interleukin (IL)-6. As a result, the pro-inflammatory CAAs exhibit an increased expression of IL-6 and a proliferation-promoting effect on breast cancer cells [141]. During ovarian cancer metastatic progression, omental CAAs and CAFs deliver miR-21-containing exosomes to cancer cells. Mir-21 targets apoptotic protease activating factor 1, inhibits ovarian cancer apoptosis, and confers paclitaxel chemoresistance [142], suggesting stromal-derived miR-21 blockade as a potential therapeutic strategy for metastatic and refractory ovarian cancer. Profiling of miRNAs in tumor interstitial fluid of human breast tumor tissues identified a list of 23 miRNAs that were associated with the presence of adipocytes and immune cells in tissues, suggesting epigenetic tumor-stroma crosstalk [143].

A genome-wide expression profiling of peri-prostate adipose tissue samples taken from prostate cancer patients revealed shifted expression of genes collectively accounting for increased proliferative and anti-apoptotic activity and mitigated immunosurveillance, suggesting that the peri-prostate adipose tissue cultivates prostate cancer development [144]. A related epigenome-wide DNA methylation pilot study of peri-prostate adipose tissue in normal weight and obese prostate cancer patients, performed by the same research group [145], revealed abundant DNA hypermethylation in cancer patients with excessive adiposity, with the epigenetically altered genes contributing to altered fatty acid metabolism, immune perturbations (including those providing tumor immune evasion), pluripotency of stem cells, and other pathways that are advantageous for cancer support. Concordantly, analysis of DNA methylation in omental tissue of obese women revealed significant DNA hypermethylation, whereas subsequent weight loss had a substantial hypomethylating effect [146]. Interestingly, obesity-induced proinflammatory cytokines triggered expression of DNMT1 and methylation of adipokine gene, whereas treatment with the DNMT inhibitor reverted the process in adipocytes [147]. Recent work by Tang et al. [148] demonstrated that treatment of adipocytes alone with the DNMT inhibitor resulted in the re-expression of tumor suppressor genes (e.g., *SUSD2*, *TFP12*, *GREM1*, *TRIM29*), altered expression of EMT mediators (e.g., *CDH1*, *CDH2*, *FN1*, and *SLUG*), and diminished migrative and invasive properties of co-cultured EOC cells. These data provide clear evidence that therapeutic tailoring of epigenetic aberrations in CAAs may, in turn, have anti-metastatic effect on ovarian malignant cell behavior [148].

Taking into consideration that discovery of lncRNAs as a class occurred very recently, there is an ample gap in evidence of their regulatory role in tumor-stroma interplay. However, recent studies report that lncRNAs are capable of mediating the expression of genes that are associated with lipid adipogenesis and metabolism via RNA, DNA or miRNA complementation, or recruitment of proteins involved in modulation of histone markers [149,150,151]. A recent study, including transcriptome profiling of primary brown and white adipocytes, preadipocytes, and cultured adipocytes reported a list of 175 lncRNAs that are specifically regulated during adipogenesis [152]. Given a constantly increasing number of lncRNAs with documented significance in the development of a variety of cancers, including ovarian [94,153,154], investigation of lncRNA-associated epigenetic modifications in the context of interrelationship between EOC and CAAs will undoubtedly be a very fruitful and promising direction for further basic/translational research.

### 2.3. Mesenchymal Stem Cells (MSCs)

MSCs encompass a diverse multipotent cell subgroup that was recruited to the tumor stroma from such sources as bone marrow, adipose tissue, umbilical cord, endometrium (menstrual blood), pericytes, as well as other organ-specific locations. The existence of MSCs has been reported for the majority of organs and tissues, including ovary (Figure 1A) [155]. Due to their capacity to differentiate into other active pro-cancerous stromal components (such as CAFs and CAAs) as well as sustain a cancer stem cell (CSC) population, MSCs are strongly associated with cancer progression yet retain a normal (non-malignant) genotype. In particular, ovarian carcinoma-associated MSCs (CA-MSCs) exhibit multipotent potential and strong EOC growth-permissive and stemness-promoting properties, increasing resistance to platinum-based chemotherapy, providing tumor stromal support and neovascularization [156,157,158,159,160,161]. Contradictory to those reports are studies demonstrating tumor-restricting effect of human MSCs on EOC cells via stimulation of EOC cell cycle arrest, enhanced apoptosis, altered mitochondria membrane potential and suppressed neoangiogenesis [162], and the inhibition of cisplatin-resistant ovarian carcinoma xenograft growth [163]. Given a wealth of controversial data on the assisting vs restricting role of MSCs on EOC development and the extensive interest in therapeutic applications of MSCs as vehicles for EOC-targeted drug delivery due to their high tumor site tropism [164,165,166,167,168], further investigations to determine the underlying mechanisms of MSC-EOC interactions are clearly necessary.

While the overwhelming majority of research work is focused on the elucidation of cell signaling pathway implications, the epigenetic interplay between MSCs and EOC remains largely unexplored. While considering the well accepted role of epigenetic aberrations in CSC reprogramming (comprehensively outlined in [169]), emerging evidence on efficient epigenetic transformation of MSCs into CSCs via tailoring chromatin remodeling (imbalanced DNA methylation of cancer-implicated genes, application/loss of histone marks, etc.) [170,171], and epigenetic modulation of MSC differentiation into diverse stromal cell lineages [172,173,174], the involvement of EOC-mediated epigenetic factors on MSC phenotype and functioning, and the subsequent reciprocal effect of reprogrammed MSCs on ovarian carcinoma, is highly plausible. Toward these possibilities, Reza et al. [175] reported the anti-proliferative and pro-apoptotic effect of adipose-derived MSC exosomes on ovarian cancer A2780 and SKOV3 cells; subsequent exosomal RNA sequencing identified a list of enriched miRNAs targeting EOC cell survival pathway genes [175]. MiRNA expression profiling in aging MSCs revealed altered expression of two miRNAs, miR-638 and miR-572, both of which have been reported to be upregulated in ovarian carcinoma; however, distinct studies display controversial findings on the impact of these miRNAs on EOC [176,177,178,179]. Among other non-coding RNAs, abundant in MSC-derived extracellular vesicles and associated with ovarian cancer development are miR-21, miR-92a and miR-221 [180]. Alternatively, Ho and colleagues [181] demonstrated, that ovarian cancer stromal progenitor cells isolated from tumor tissues and ascitic effusions of EOC patients displayed 40 hypermethylated tumor suppressor genes (TSGs) (with DLC1, RASS382, CDH13, BRCA1, TIMP3, HIN-1, ESR1, CDKN2A, CCND2, CDKN2B, as most frequently hypermethylated and correlating with validated mRNA expression decrease in DLC1, RASSF1A, CCND2, and CDKN2B) in comparison with matching patient ovarian cancer cells and were capable of promoting tumor growth in vivo when co-injected with SKOV3 cells. Most importantly, treatment with the hypomethylating agent (HMA) 5-aza-2-deoxycytidine (decitabine or DAC, a desoxyribonucloside that exclusively incorporates into DNA and inhibits DNMTs by irreversible binding of the latter [182]) resulted in efficient demethylation of TSGs in stromal progenitor cells, repressed their self-renewal and growth and mitigated proliferation of ovarian tumor cells [181]. Collectively, these studies suggest a direct or indirect epigenetic relationship between MSCs and ovarian tumor cells and warrant additional research that can potentially lead to the identification of novel therapeutic targets for ovarian carcinomas.

### 2.4. Tumor-Associated Endothelial Cells (TECs)

TECs refer to endothelial cells that line the inner walls of newly formed blood vessels in tumors. TECs support blood flow, tumor tissue trophics, and accelerate tumor progression. TECs are genetically non-malignant cells, however, they differ from normal endothelial cells by exhibiting cytogenetic abnormalities [183], distorted morphology [184], and altered molecular profiles [185], as well as improper functional characteristics. TECs form a disorganized, excessively sprouted, branched, fragile, and gap-prone endothelial network, which allows for chaotic non-laminar blood flow, increased vascular permeability and escape of primary tumor cells into blood circulation. TECs help circulating cancer cells overcome anoikis and escort their movement to metastatic niches [186]. Finally, TECs are able to develop taxol resistance in the presence of surrounding malignant milieu [187].

TECs exhibit considerable heterogeneity (reviewed in [188]), and diverse features of TECs in various tumors are attributed to the specific malignant settings [189]. In the context of epigenetic regulation, Maishi et al. [190] implanted TECs that were isolated from the high metastatic (HM) melanomas into low metastatic (LM) melanomas and achieved metastatic enhancement as a result of elevated levels of proteoglycan biglycan, attracting tumor cells to intravasate. Strikingly, upregulation of biglycan was due to DNA demethylation of its promoter region in HM-TECs as opposed to normal endothelial cells, LM-TECs, and tumor cells [190]. Furthermore, exposure of LM-TECs to conditioned medium from HM-tumors and treatment with the HMA decitabine proved the upregulation of biglycan in LM-TECs via DNA demethylation triggered in the presence of HM-tumor [190]. The study elegantly reaffirmed epigenetically-mediated bidirectional communication between tumor cells and TECs, and further suggested epigenetic perturbations as attractive targets to manipulate neoangiogenesis and tumor metastasis.

Epigenetic regulation of TECs has been reported. DNMT and HDAC inhibitors efficiently mitigated TEC growth in vitro and in vivo [191,192]. Moreover, epigenetic modifications were shown to regulate TEC-mediated immune response [193]. Downregulation of intercellular adhesion molecule-1 (ICAM-1) in TECs due to promoter histone H3 deacetylation and the loss of histone H3 Lysine^4^ methylation was reported, and re-expression of ICAM-1 in TECs by DNMT and HDAC inhibitors successfully restored leukocyte-TEC adhesion and leukocyte infiltration of vessel walls in vitro and in vivo, respectively [193]. In a separate study, the same group identified additional anti-angiogenesis genes (including clusterin, fibrillin 1, and quiescin Q6) downregulated in normal endothelial cells subjected to a tumor-mimicking setting (conditioned medium) via epigenetic silencing by promoter histone H3 deacetylation and loss of histone H3 Lysine^4^ methylation [194]. Furthermore, subsequent treatment with DNMT and HDAC inhibitors reversed the gene silencing effects [194].

An influential study assessing the role of EOC on TECs was conducted by Sood group [195], who performed a comparative genome-wide gene expression analysis of endothelial cells from five different normal ovarian tissues and TECs isolated from 10 invasive ovarian carcinomas and revealed a list of 400 differentially expressed genes, among which EZH2 was significantly upregulated. EZH2, a histone-lysine N-methyltransferase, is frequently overexpressed in ovarian tumor tissues and is a well-established epigenetic mediator (stimulator) of EOC tumor growth, invasion, metastasis, neoangiogenesis, platinum-resistance and ovarian cancer stem cell renewal through transcriptional repression of signaling pathways, as well as direct/indirect interaction with multiple miRNAs and lncRNAs (reviewed in [196]). Overexpression of EZH2 in TECs by the surrounding EOC setting occurred in a VEGF-mediated paracrine manner, which in turn promotes EZH2 direct binding, methylation, and transcriptional repression of vasohibin-1 (a selective negative regulator of TEC migration, proliferation, and vessel tube formation), stimulating further angiogenesis [197]. Remarkably, the silencing of the EZH2 gene in vitro and in vivo (via systemic siRNA nanoparticle delivery in both EOC cells and TECs in mouse EOC xenografts) substantially increased vasohibin-1 expression and reduced neoangiogenesis and tumor growth [197], underlining the additional therapeutic potential of concomitant EZH2 targeting in TECs and EOCs. Noteworthy, the expression of EZH2 in ovarian cancers has been shown to be regulated by different non-coding RNAs, for example miR-298 [198] and lncRNA HOTAIR (HOX transcript antisense RNA) [199], providing additional druggable epigenetic targets in EOC cells and TECs in order to suppress neoangiogenesis and ovarian tumor progression.

### 2.5. Pericytes

Another type of vessel-associated cells, pericytes (Figure 1), play a key role in normal vasculature development, including neoangiogenesis, and in cancer contribute to tumor development and metastasis. These perivascular cells reside in the microvessel walls, immediately beyond the basement membrane, and provide physical support to endothelial cells, control integrity of the vessel endothelial layer, vascular permeability, and blood flow [200], serve as an origin of mesenchymal stem cells (MSCs) for some human tissues [201] and as multipotent precursors for other types of cells [202], and exert immune mediators [203]. During cancer progression, pericytes essentially contribute to rapid neoangiogenesis and tumor growth, expansion of the CSC population, on one hand, but also strengthen immune defense against tumor invading and restrict metastatic seeding, on the other hand, as comprehensively outlined in [204,205]. In EOC patients, high pericyte score (carrying a pericyte gene signature) was shown to be an unequivocal predictor of earlier relapse and poor prognosis [206]. Dual targeting of pericytes in combination with endothelial cells [207], as well as the disruption of connections between pericytes and endothelial cells via insertion of N-cadherin blocking peptides [208], are potential anti-angiogenesis therapeutic strategies in EOC.

Intriguingly, in a HGSOC xenograft model, injection of pericytes concomitantly with EOC cells amplified tumor growth and metastasis rate without altering tumor vasculature, highlighting a direct impact of perycites on EOC cells, independent of neoangiogenesis [206]. No studies have adequately described epigenetic tumor-perycite crosstalk in ovarian carcinomas. However, proximity of the malignant (glioblastoma) setting [209], hypoxic conditions [210] and inflammatory stimulation [211] trigger an epigenetically mediated (through altered non-coding RNA) pericyte response. Though these findings are quite limited, they raise the possibility of dynamic epigenetic cooperation between pericytes and EOC cells (and other cancer-associated stromal cells) within a highly reactive, proinfammatory, and hypoxia-prone ascitic TME. In addition, a very interesting preclinical study by Kratzsch et al. [212] assessed the effect of 5-azacitidine (AZA; a ribonucleoside that is capable to incorporate into cellular RNA and DNA and acts as a HMA interfering with the DNMT activity [182,213]), valproic acid (HDAC inhibitor), temozolomide (a standard DNA alkylating chemotherapeutic positive control), and a bevacizumab (an angiogenesis inhibitor targeted therapy positive control) on the tumor growth and neovasculature status in the murine glioblastoma multiforme models. Strikingly, besides the suppressive effect on glioma tumor growth, the HMA also resulted in a notable antiangiogenic effect via the substantial diminishing of endothelial cells and the decreasing number of pericytes [212]. Contrarily, HDAC inhibitor valproic acid had no mitigating effect either on tumor growth or on the vasculature [212]. These results suggest concordant epigenetic expression changes taking place within the malignant tissue and the adjacent TME cells as a result of their mutual communication (as both tumorous and angiogenesis cells selectively responded to the same epigenetic-tailoring drug, but not to the other). In dissonance with that is work by Karén et al. [214], who reported successful inhibition of pericyte differentiation, migration, and proliferation in response to HDAC inhibitors in vitro, and HDAC suppression stimulated expression of genes regulating vessel stabilization and maturation in pericytes. Collectively, these data highlight translational potential of cancer-prone epigenetic signatures not only in the neoplastic component *per se*, but in the surrounding stroma as well.

### 2.6. Tumor-Associated Macrophages (TAMs)

TAMs within malignant stroma (Figure 1A) are considered to be a fundamental immune cell subpopulation responsible for cancer-associated inflammation, matrix remodeling, tumor immune escape, growth, invasion, angiogenesis, metastasis, cancer cell stemness, and drug resistance. Macrophage diversity and ability to transfer between M1 (classic, host immune defense activating, tumoricidal) and M2 (alternative, immunosuppressive, pro-tumorigenic) phenotypes are defined by the distinct tumor microenvironments [215]. Several recent reviews [216,217] elegantly describe bidirectional TAM interactions with cancer cells, other immune cell subpopulations and non-malignant stromal components, such as CAFs, TECs, B cells, eosinophils, basophils, dendritic cells, natural killer cells, and others.

In addition to ample evidence on receptor-ligand signaling interrelationship between EOC and TAMs [218,219,220,221,222,223], a better understanding of their epigenetic cooperation has begun to accrue. Ying et al. [224] reported initiation of M2 polarization and tumor-promoting capabilities in ovarian TAMs by EOC-released exosomal miR-222-3p through SOCS3/STAT3 pathway. Similarly, macrophage M2 phenotype shift was induced by exosomal miR-940 released from hypoxic epithelial ovarian tumors [225]. Importantly, TAM-secreted exosomes suppress endothelial cell migration by targeting miR-146b-5p/TRAF6/NF-κB/MMP2 pathway, whereas the EOC-released exosomal delivery of lncRNAs to the site efficiently restores the endothelial cell movement [226]. Alternatively, Hu et al. [227] observed altered expression of 19 miRNAs in TAMs exposed to tumor necrosis factor-related inducer of apoptosis (TWEAK; commonly expressed by immune cells, such as dendritic cells and natural killer cells) and demonstrated that the exosomal transportation of overexpressed miRNAs, and in particular, miR-7, from TAMs to EOC cells significantly repressed their metastatic activity in vitro and in vivo via repression of EGFR/AKT/ERK1/2 pathway. Furthermore, the insertion of antagomiR-7 into TAMs repressed levels of miR-7 in TAMs, in released exosome vesicles and in the recipient ovarian malignant cells, and stimulated EOC metastasis [227].

Chromatin remodeling-related epigenetic modifications resulting from TAM-EOC interaction remain largely unknown. However, as reported for gastric cancer, TAMs are capable of silencing TSG gelsolin in cancer cells by increased DNMT1 expression and DNA methylation of gelsolin promoter via activation of CCL5/CCR5/STAT3 signaling [228]. Most importantly, either suppressing DNMT enzyme activity, or treatment with demethylation agent, or interfering with CCL5/CCR5/STAT3 pathway led to decreased in vivo tumor growth, suggesting several possible routes of epigenetic inhibition of gastric cancer development [228]. In oral cancer, interferon-γ mRNA expression in TAMs was substantially decreased in comparison with normal or benign oral tumor specimens as a result of promoter region methylation, with the level of methylation strongly correlating with the clinical stage, histopathology grade, and primary tumor scale [229]. Promoter hypermethylation and downregulation of the follistatin like-1 (FSTL-1) glycoprotein was associated with metastatic activity of nasopharyngeal carcinomas and dysfunction of macrophages, whereas treatment with recombinant human FSTL-1 protein elevated IL-1β and tumor necrosis factor-α in TAMs and repressed cancer cell immune evasion [230]. Using a panel of distinct cancer type cells, including cervical, hepatocellular, epidermoid carcinomas, glioblastoma, rabdomyosarcoma, and murine melanoma, Osawa and co-authors [231] demonstrated that cancer cell hypoxia and nutrient starvation lead to activation of histone demethylase JMJD1A (Jumonji domain-containing 1A), followed by increased AKT phosphorylation, cancer cell metastatic properties, increased angiogenesis, and infiltration of macrophages into cervical cancer and muscle sarcoma tissues in vivo. Remarkably, JMJD1A repression mitigated tumor progression through decreased neovascularization and alleviated TAM infiltration, and importantly, enhanced the anti-tumor effect of anti-angiogenesis agents bevacizumab and sunitinib [231]. Ishii et al. [232] described M2 macrophage polarization via reciprocal epigenetic changes in histone H3 lysine^4^ and histone H3 lysine^27^ methylation through STAT6 mediation, which collectively lead to transcriptional activation of specific M2 maker genes. Altogether, these findings underline the immediate importance of continued research accessing TAM-modulated epigenetic changes in EOC, as they may reveal novel diagnostic and therapeutic approaches to mitigate TAM-associated pro-tumoral inflammation and cancer progression, and boost host immune defense mechanisms.

### 2.7. Tumor-Infiltrating Lymphocytes (TILs)

TILs are collectively represented by varying amounts of T and B lymphocytes recruited from the circulatory system to the tumor site to fulfill the host immune response function. In EOC, TILs may be present in primary tumors and advanced metastatic lesions, both within malignant (intratumoral) bulk and stromal compartment, and differential representation of certain TIL subsets (helpers, killers, regulatory/suppressors, effectors, memory cells, and such) depends on the disease stage, therapeutic management, chemotherapy response status, and possesses prognostic significance (Figure 1A) [29,233,234,235,236,237,238].

The extensive evidence on signaling molecules and pathways implicated in TIL functioning and ovarian cancer prognosis, as well as current attempts to differentially manipulate TIL subsets and signaling networks towards boosting adequate anti-tumor immunity, have been comprehensively summarized by Santoiemma and Powell in a recent review [239]. The epigenetic regulation of TIL functioning in ovarian cancer and epigenetic-based immunotherapeutic strategies are emerging areas of interest. Sehouli et al. [240] proposed the concept of “epigenetic immunophenotyping” (identification of certain epigenetic marks) of both overall and specialized TIL populations. By using matching healthy and cancer tissues, including ovarian, they established a strong correlation between epigenetics and cancer prognosis [240]. In support of this concept, treatment with DNMT inhibitor 5-azacitidine led to the substantial enrichment for immunomodulatory pathways (interferon signaling, antigen processing and presentation, and cytokines/chemokines) in ovarian and other cancers [241]. Similarly, with the use of global gene expression profiling of EOC treated with DNMT inhibitor decitabine, Wang et al. [242] discovered prominent upregulation of immunoregulatory genes cohort in decitabine-exposed malignant cells. The group further showed that decitabine treatment stimulated TIL infiltration and anti-tumor function in both subcutaneous and intraperitoneal syngeneic murine ovarian cancer models [242]. Moreover, combining a DNMT inhibitor with the standard immune checkpoint blockade antibody stimulated conversion of naïve T cells into T effectors and contributed to better mouse survival [242]. Based on this work, Stone et al. [243] further demonstrated that the activation of type I interferon signaling in response to DNMT inhibitor 5-azacytidine was a key requirement for efficient stimulation of CD45^+^ (leukocyte common antigen) immune cells, CD8^+^ cytotoxic T cells, natural killer cells, restriction of macrophages, and myeloid-derived suppressor cells in the ovarian TME in vivo, prompting the inhibition of tumor growth and increased survival. In support, Adair and Hogan [244] demonstrated the enhanced expression of cancer-testis antigens and class I major histocompatibility complex (MHC)-encoded molecules in EOC cells that were treated with DNMT inhibitors and subsequent infiltration of antigen-reactive CD8^+^ cytotoxic T cells to EOC. Consistent with that, seminal work by Chiappinelli and co-authors [245] established that DNMT inhibitors activate interferon signaling, TIL infiltration, and EOC cell death via the upregulation of viral defense pathway, as hypomethylation of endogenous retrovirus (ERV) genes leads to upregulated viral defense gene expression and boosts immune response.

In the context of non-coding RNA interference, two prognostic miRNA signatures—malignancy signature and immunological signature—have been recently identified in advanced EOC [246]. Briefly, using integrative mRNA/miRNA co-expression analysis of primary tumor tissues from advanced EOC patients, two modules were established: a malignancy module (let-7f, let-7g, miR-106a, miR-17, miR200c, miR-26a, miR-26b, and miR-328), which was associated with the more aggressive EOC growth and an immunological module (miR-197, miR-22, miR-22#, miR-28, miR-339–5p, miR-340#, miR-628–5p, miR-629, miR-661, and miR-98), which strongly correlated with intratumoral infiltration by T cells, natural killer cells, cytotoxic lymphocytes, and macrophages [246]. These microRNA signatures may serve as prognostic and treatment efficacy biomarkers as well as potential targets for epigenetic-based immunotherapy of advanced ovarian cancer [246].

### 2.8. Plasmacytoid Dendritic Cells (PDCs)

PDCs constitute a rare, yet critically important and highly specialized immune cell subpopulation whose main role in immune surveillance is rapid recognition of foreign pathogens via selectively expressed toll-like receptors and the immediate activation of both innate and acquired immune systems (Figure 1A) [247]. Incessant stimulation of PDCs by self-DNA (a situation when PDCs, which do not normally react to inert DNA of organism’s cells, become continually activated by the altered DNA of transformed cells) is a characteristic of a variety of neoplasms, including ovarian carcinomas [248,249,250]. Accumulation of PDCs within the epithelial ovarian tumor bulk promotes vasculogenesis [251,252] and immune tolerance [253], and it is associated with unfavorable disease prognosis [254]. Importantly, the elimination of immature PDCs in mice bearing ovarian tumors via the targeting of specific markers led to vascular ablation, substantially reduced tumor growth, and triggered anti-tumor immune response and tumor re-sensitization to chemotherapeutic agents [255].

Epigenetic regulation of hematopoietic stem cell lineage commitment and subsequent differentiation of dendritic cell precursors into specific dendritic subtypes (including PDCs) and interferon response in the context of chromatin remodeling, miRNA and lncRNAs interference are well documented [256,257,258,259,260] and not focused on herein. Meanwhile to our knowledge, the epigenetic impact on and by PDCs in relation to EOC remains largely undefined. A single study demonstrated that exogenous supplementation of miR-155 (which is downregulated in tumor-associated PDCs) via targeted nanoparticle delivery to EOC-associated PDCs results in the genome-wide silencing of multiple immunosuppressive mediators, following stimulation of immune defense mechanisms and abolishment of ovarian carcinoma progression in vivo [261]. Importantly, a comparison of methylome profiles of dendritic cells and myeloid-derived suppressor cells (MDSCs, an immune cell subtype bearing same progenitor with PDCs) grown under tumor-associated conditions (exposure to prostaglandin E2 or malignant conditioned medium) revealed MDSC-specific DNMT3A enzyme activation, DNA methylation signature, and silencing of immunogenic-associated genes analogous to changes that were observed in primary MDSCs dissected from ovarian cancer patients [262]. Moreover, the suppression of DNMT3A activity abrogates MDSC-specific hypermethylation and MDSC immunosuppressive properties [262]. These findings support the epigenetic tuning of immune surveillance by ovarian TME and underline the necessity for further investigations in this direction.

### 2.9. Mesothelial Cells (MCs)

MCs are simple squamous epithelial cells that line the intra-abdominal cavity as an upper single layer (mesothelium) of the peritoneum, immediately supported by the basal membrane, underneath which is the collagen-rich extracellular matrix (Figure 1A). MCs function as an active physical barrier against the invasion of ovarian neoplastic cells into submesothelial matrix and metastasis formation, and mesothelial disruption (clearance) imposes a higher level of EOC peritoneal adhesion and dispersal [263,264]. Besides, MCs serve a secretory role by releasing bioactive soluble factors into malignant ascites, such as phospholipid lysophosphatidic acid [265] and VEGF [266], which are known to enhance EOC tumor growth, metastasis, apoptotic resistance, and angiogenesis [265,267,268,269]. MCs also produce large amounts of tumor-associated immunostimulatory protein K90 [270], associated with the poor prognosis in ovarian and breast cancers [271,272] and implicated in drug resistance [273].

The bidirectional interplay between EOC cells and MCs has been recently assessed in a study by Matte et al. [274], who reported global gene expression changes (649 altered genes primarily related to cell growth and proliferation, apoptosis, cell cycle, cell assembly, and organization) in MCs exposed to EOC ascitic effusions [274]; reciprocally, EOC cells exposed to EOC-associated MC setting exhibited an enhanced resistance to induced apoptosis [274]. However, epigenetic regulation underlying these gene expression alterations are unknown. Clearly, DNA methylation, histone modification profiling, and non-coding RNA profiling are further key steps necessary to distinguish specific epigenetic mechanisms responsible for gene expression switches occurring in EOC cells and cancer-affected mesothelium because of their interdependence. Gaining better understanding of these events may provide valuable insight on cellular adjustment during tumor-mesothelial adhesion step and suggest novel approaches to block metastatic seeding of the peritoneum.

## 3. Ovarian TME: Potential Epigenomic-Based Therapeutic Strategies

**Figure 3:**
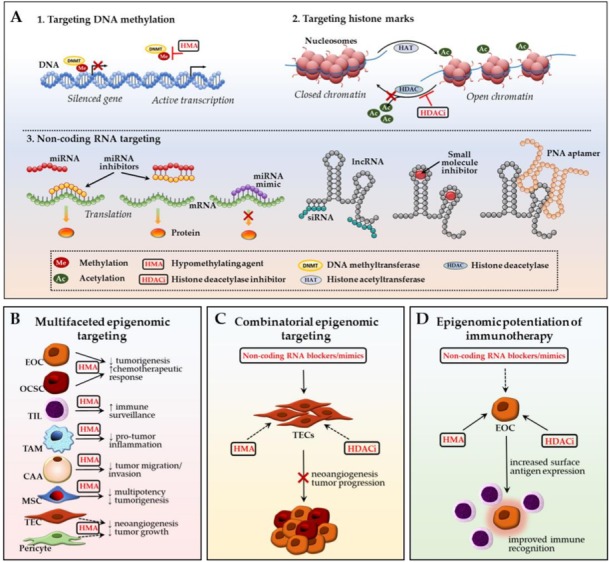
Ovarian TME: potential epigenomic-based therapeutic strategies. (**A**) Main epigenetic therapy mechanisms include: (1) DNA demethylation with hypomethylating agents (HMAs) to re-activate transcription of silenced genes; (2) restoration of open, transcription-permissive euchromatin state with histone deacetylase (HDAC) inhibitors preventing removal of acetyl groups from the histone tails; (3) targeting non-coding RNA-mediated epigenetic perturbations via delivery of exogenous miRNA inhibitors (antagomirs/blockmirs/sponges) or mimics, lncRNA siRNAs, small molecules or peptide nucleic acid aptamers; (**B**) Multifaceted epigenomic targeting approach suggests simultaneous targeting of several malignant and TME cell types using one epigenetic drug; (**C**) Combinatorial epigenomic targeting involves employment of multiple epigenetic mechanisms to revert cancer-associated phenotype in TME cells and inhibit their tumor-promoting effect on EOC cells; (**D**) Epigenomic potentiation of immunotherapy involves epigenetic stimulation of cancer cell immune gene/pathway representation which allows for increased immune surveillance efficacy. CAA—cancer-associated adipocyte; HMA—hypomethylating agent; HDACi—histone deacetylase inhibitor; EOC—epithelial ovarian cancer cell; MSC—mesenchymal stem cell; OCSC—ovarian cancer stem cell; PNA—peptide nucleic acid; TAM—tumor-associated macrophage; TEC—tumor-associated endothelial cell; TIL—tumor-infiltrating lymphocyte. Black arrows represent interactions reported in EOC; dashed arrows designate patterns that were observed in other cancer types and may potentially be applicable towards ovarian cancer (see main text for details).

The growing body of information on epigenetic control of ovarian cancer metastatic advancement and acquisition of resistance to standard-of-care chemotherapy make epigenomic alterations attractive prognostic markers and druggable targets to improve therapeutic outcomes in EOC patients especially with platinum-resistant and recurrent disease. Our group and others are actively exploiting DNA hypermethylation, modifications of histone marks, and aberrant expression of non-coding RNAs in malignant EOC cells in an attempt to treat EOC, re-sensitize ovarian tumors to conventional chemotherapy, and prevent/delay disease recurrence (Figure 3A). We and others aim to discern global DNA methylation patterns and methylation states of specific candidate genes to prognosticate and improve patient response to chemotherapies with hypomethylating agents (HMAs) [15,88,275,276,277]. We recently demonstrated successful in vitro and in vivo re-sensitization of multiple cisplatin-resistant ovarian cancer cell lines using a novel small-molecule DNMT inhibitor guadecitabine (SGI-110) [275]. Efficient epigenetic targeting of ovarian CSC population by the HMA with induction of differentiation, restriction of tumor-initiating capacity, and re-sensitization to platinum was also reported [278]. In a recently completed phase I clinical trial, guadecitabine “priming” in combination with carboplatin induced clinical response in patients with platinum-resistant, recurrent HGSOC [279]. Similarly, HDAC inhibitors are being examined in advanced, refractory, and recurrent ovarian cancer [280,281,282]. However, despite some promising results, cell adaptation to HDAC inhibitor-mediated epigenomic disruption has been frequently observed [283,284,285]. Finally, the recent identification of large miRNA and lncRNA classes as epigenetic regulators of gene expression granted new opportunities for miRNA- and lncRNA-employed prognostic evaluation and targeting of EOC [92,93,94,153,286].

Continuous accumulation of knowledge on epigenomic perturbations in ovarian TME cellular compartments in the context of their communication with EOC cells holds great translational promise. The fact that non-malignant stromal cells lack genetic mutations and acquire potentially reversible tumor-specific molecular traits and cancer-indulgent functions via epigenetic reprogramming by EOC cells suggests the epigenetic tailoring of ovarian TME as a promising strategy in ovarian cancer management. Given the varying stroma representation in ovarian malignancies (stromal compartment may range from 7 to 83% in different ovarian tumors [287]) and high level of TME cell heterogeneity, it is pivotal to correctly evaluate TME composition within the tumor bulk in each disease case. Prevalence of one or another type of TME cells may dictate activation of certain mechanistic pathways, help to dissect “first choice” epigenetic therapy candidates, and possibly lead to the identification of epigenetic alterations in the TME that are specific for each patient.

Treatment of different stromal components with HMAs demonstrated tumor-restrictive potential. In particular, the exposure of adipocytes to guadecitabine attenuated HGSOC cells metastasis-associated behaviors [148]. HMA decitabine effectively inhibited the multipotent and tumor-promoting capabilities of ovarian cancer-associated MSCs and led to decrease in cancer cell proliferation [181]. While not specified for EOC, treatment with the DNMT inhibitor azacitidine showed tumor-inhibiting and anti-angiogenesis effect with a decreased amount of TECs and pericytes in other cancer types [212]. HMA therapy also showed change in immunoregulatory cell response within the tumor bulk with increased infiltration of tumor-restrictive TIL subsets and suppression of TAM-mediated tumor progression in EOC and other cancers [228,242,243,244]. Such multifaceted epigenomic targeting of multiple stromal cell types in parallel with primary EOC cell targeting through assessing the same epigenetic mechanism (Figure 3B) may improve efficacy, while also diminishing the toxic effects that are associated with multi-drug treatment regimens.

Alternatively, similarly to an improved anticancer effect of a DNMT/HDAC inhibitor combination on ovarian tumors [288] and other malignancies [284,289], the combinatorial epigenomic targeting of ovarian TME components using several epigenomic approaches is plausible (Figure 3C). For example, anti-angiogenesis and anti-proliferative effect of simultaneous utilization of an HDAC inhibitor to target pro-tumorigenic histone marks and an HMA to demethylate DNA promoter CpG islands in colon tumor-conditioned TECs was reported [194]. Analogous drug combination may have therapeutic prospective to block ovarian cancer neovascularization. Moreover, systemic nanoparticle delivery of siRNAs or antagomirs targeting tumor-assisting non-coding RNAs (such as EZH2, HOTAIR, and miR-298) into ovarian TECs may provide an additional option for combinatorial blocking of EOC tumor growth and angiogenesis [197,198,199]. Current data provide strong rationale for combining HDAC and DNMT inhibitors to affect EOC-associated pericytes [212,214].

Given that CAF reprogramming is shown to repress ovarian tumor advancement [290], prevention or reversion of CAF phenotype may potentially be accomplished through combined treatment with a HMA [113,115] and a CAF-delivered mixture of miRNA miR-31/miR-214 mimics plus miR-155 antagonist to interfere with the EOC-associated CAF miRNA signature [130]. The recent discovery of a number of altered lncRNAs in HGSOC CAFs [132] may suggest novel options for restricting CAF-promoted EOC growth via targeted siRNA injections. Similarly, non-coding RNA signatures recently identified in EOC-related immune cell populations (discussed earlier in this review) warrant further siRNA/miRNAs targeted conveyance studies in this direction. Importantly, in contrast to CAFs, miR-155 was downregulated in EOC-associated PDCs and its exogenous delivery to these cells, in turn, boosted anti-tumor immune defense response and EOC suppression [261]. Taking into consideration such drastically opposite effects of the same epigenomic regulators on different ovarian TME cell compartments, the development of methodologies for highly specific nanoparticle transportation and precise cell type targeting is pivotal. Several studies investigating the potential of exosome-facilitated epigenomic (miRNA) cargo delivery for EOC treatment are also in progress [291].

Exploiting host immune system in managing ovarian cancer growth and metastatic spread has emerged as another key research and therapeutic direction [292,293,294,295,296]. A number of comprehensive analyses of epigenomic strategies employed to advance cancer immunotherapies [297,298,299] provide detailed illustration on how currently available epigenetic drugs may potentially contribute to modulation of cancer immunosurveillance and anti-tumor responses (Figure 3D). In ovarian cancer in particular, Stone et al. [243] demonstrated while using preclinical in vivo models that combining DNMT inhibitor and HDAC inhibitor with immune checkpoint blockade resulted in the most notable anti-cancer effect and survival due to activation of type I interferon signaling, more efficient recruitment of anti-tumor TIL subsets and the restriction of tumor-indulgent TAMs and myeloid-derived suppressor cells to the ovarian TME. Alternatively, Song and coworkers [300] reported HDAC inhibitor-stimulated increase of NKG2D ligand expression on the surface of ovarian cancer cells, which allowed for better recognition and killing of the latter by engineered NGK2DL-specific chimeric antigen receptor T cells. Finally, Wargo and colleagues [301] reported improved recognition of tumor cells by engineered peripheral blood lymphocytes in response to increased cancer-testis antigen NY-ESO-1 expression (typically expressed in the majority of epithelial tumors, including ovarian [302]), catalyzed by DNMT inhibitor (alone or combined with HDAC inhibitor). Furthermore, the results of a recent phase I clinical trial by Odunsi et al. [303] showed improved efficacy of NY-ESO-1 vaccine therapy when combined with escalated doses of HMA decitabine in patients with EOC relapse. Current clinical trials combining immunotherapeutic approach with epigenetic drug(s) are summarized in Table 1. In addition to epigenomic potentiation of immunotherapy by chromatin remodelers, boosting regulatory T cell-mediated immune response may be achieved via targeting non-coding RNAs [304] and it requires further testing for applicability in EOC (Figure 3D).

## 4. Conclusions

To summarize, a deeper understanding of epigenomic involvement in reciprocal EOC tumor-stroma interrelationship is essential and it will help to determine pharmacological routes to alter the TME, which in turn could inhibit EOC metastatic progression and the development of chemoresistance and tumor recurrence. Furthermore, we believe epigenetically-mediated pharmacological engagement of certain TME players, such as immune cells, to boost immune responses towards complete malignant cell elimination has tremendous potential.

## Figures and Tables

**Table 1:** Clinical trials evaluating safety and efficacy of immunotherapeutic agents in combination with epigenetic drugs in patients with ovarian cancer (https://clinicaltrials.gov).

Study Name	Phase	Status	ClinicalTrials.gov Identifier
Decitabine, Vaccine Therapy, and Pegylated Liposomal Doxorubicin Hydrochloride in Treating Patients With Recurrent Ovarian Epithelial Cancer, Fallopian Tube Cancer, or Peritoneal Cancer	I	Completed Completion: June 2013	NCT01673217
Atezolizumab, Guadecitabine, and CDX-1401 Vaccine in Treating Patients With Recurrent Ovarian, Fallopian Tube, or Primary Peritoneal Cancer	I/IIb	Ongoing Patient recruitment suspended Estimated completion: March 2020	NCT03206047
Guadecitabine and Pembrolizumab in Treating Patients With Recurrent Ovarian, Primary Peritoneal, or Fallopian Tube Cancer	II	Ongoing Recruiting patients Estimated completion: March 2020	NCT02901899
Genetically Modified T Cells and Decitabine in Treating Patients With Recurrent or Refractory Ovarian, Primary Peritoneal, or Fallopian Tube Cancer	I	Ongoing Recruiting patients Estimated completion: August 2020	NCT03017131
Study of Azacitidine and Durvalumab in Advanced Solid Tumors (METADUR)	II	Ongoing Recruiting patients Estimated completion: January 2022	NCT02811497
